# Gadolinium-conjugated star-block copolymer polylysine-modified polyethylenimine as high-performance *T*_1_ MR imaging blood pool contrast agents†

**DOI:** 10.1039/c7ra08820e

**Published:** 2018-01-30

**Authors:** Zhongjie Huang, Yicun Chen, Daojun Liu, Chao Lu, Zhiwei Shen, Shuping Zhong, Ganggang Shi

**Affiliations:** Department of Radiology, The First Affiliated Hospital, Shantou University Medical College Shantou 515041 China zjhuang@stu.edu.cn; Department of Pharmacology, Shantou University Medical College Shantou 515041 China chenyicun@yeah.net ggshi@stu.edu.cn; Department of Chemistry, Shantou University Medical College Shantou 515041 China Liudj@stu.edu.cn sampson020@163.com; Department of Radiology, The Second Affiliated Hospital, Shantou University Medical College Shantou 515041 China zwshen@stu.edu.cn; Department of Biochemistry and Molecular Biology, Keck School of Medicine, University of Southern California Los Angeles California 90033 USA szhong@usc.edu

## Abstract

Core–shell copolymers have received widespread attention because of their unique properties, such as suitable for surface modification and increasing the functionality. Thus, they have been increasingly used in many fields including biomedical, pharmaceutical, electronics and optics. Here, a new core–shell copolymer system was developed to synthesize potential blood pool contrast agent (CA) for magnetic resonance imaging (MRI). The novel CA with high *T*_1_ relaxivity was synthesized by conjugating gadolinium (Gd) chelators onto star-block copolymer polyethylenimine-grafted poly(l-lysine) (PEI–PLL) nanoparticles (NPs). The *T*_1_ relaxivity of PEI–PLL–DTPA–Gd NPs measured on a 7.0 T small animal MRI scanner was 8.289 mM^−1^ s^−1^, higher than that of *T*_1_ contrast agents widely used in the clinic, such as Gd–DTPA (also known as Magnevist, *r*_1_ = 4.273 mM^−1^ s^−1^). These results show that PEI–PLL–DTPA–Gd exhibits more efficient *T*_1_ MR contrast enhancement compared to Gd–DTPA. More importantly, the PEI–PLL–DTPA–Gd core–shell NPs exhibited extremely low toxicity when measured against the HepG2 cell line over a similar concentration rang of Magnevist. In *in vivo* experiments, PEI–PLL–DTPA–Gd not only displayed good *T*_1_ contrast enhancement for the abdominal aorta, but also showed prolonged blood circulation time compared with Gd–DTPA, which should enable longer acquisition time, for MR and MR angiographic images, with high resolution in clinical practice. PEI–PLL–DTPA–Gd NPs have potential to serve as high *T*_1_ relaxivity blood pool MRI CA in the clinic.

## Abstract 2

Core–shell copolymers have received widespread attention because of their unique properties, such as suitable for surface modification and increasing the functionality.

## Graphical abstract

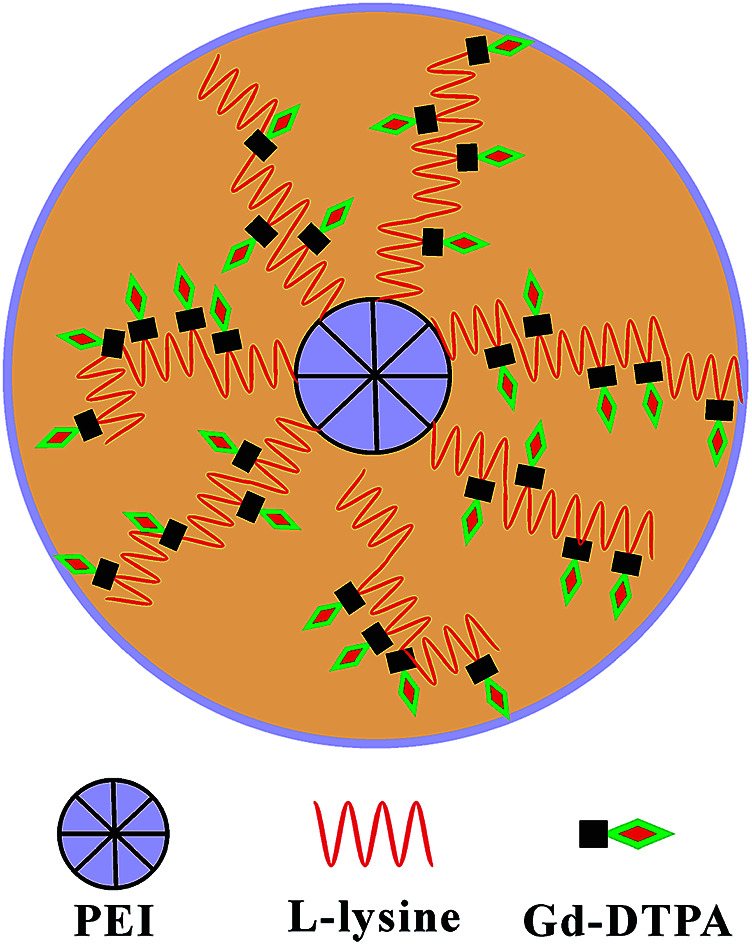

## Introduction

Magnetic resonance (MR) imaging, as one of the most versatile imaging modalities for detecting and diagnosing diseases, has unparalleled advantages such as non-invasiveness, no use of radiation, and high spatial resolution.^1^ In order to enhance the diagnostic sensitivity and specificity of MR imaging, a variety of contrast agents (CAs) have been used for contrasting normal and diseased tissues by shortening spin-lattice relaxation time *T*_1_ or spin–spin relaxation time *T*_2_ of the surrounding water protons. Generally, approved metallic contrast agents include gadolinium (Gd; ProHance, Dotarem and Magnevist), manganese (Mn; Teslascan), and iron (Fe; Endorem and Feridex).^2^ Among them, Gd-based *T*_1_ CAs are the most widely used in the clinic, and more than 10 million MRI examinations are conducted with Gd-based CAs each year.^3^ However, clinically-approved, low-molecular-weight MR imaging CAs have several shortcomings: (i) the *T*_1_ relaxation time of water protons is still relatively long when using these molecules and result in a relatively high dose of intravenously-injected contrast agent to achieve adequate signal enhancement; (ii) they have a short circulation time and (iii) they rapidly extravasate from blood vessels to the interstitial space, thus rendering them inadequate for angiographic imaging.^4^

In order to overcome the limitations of low-molecular-weight MR imaging CAs, a wide range of nano-carriers has been developed for carrying Gd^3+^, including chelates, silica, dendrimers,^5^ perfluorocarbon nanoparticles (NPs), liposomes, and micelles.^6,7^

Polyethylenimine (PEI) is not only an inexpensive reagent widely available in large quantities, but also has been frequently employed as an effective nonviral vector for gene delivery.^8^ Taking advantage of the high density of amines in its structure, PEI has also been used as a stabilizer or template to synthesize, modify, or assemble inorganic nanomaterials.^9,10^ Although amine-rich groups make PEI distinctly cytotoxic,^11^ a variety of chemical modifications, such as acetylation and PEGylation,^12,13^ can address some of these concerns and improve its biocompatibility.^14–16^

In our previous work, a novel star-block copolymer, with a branched PEI core and poly-(l-lysine) (PLL) as the outer shell, was developed.^14^ Since PEI–PLL has been proven to be non-toxic, non-antigenic, biocompatible and biodegradable, it may have potential for clinical research or applications.^17^ Moreover, PLL with lysine as a repeat unit has a large amount of amino groups, which allows for the conjugation of larger amounts of the chelating agent diethylene triamine pentaacetic acid (DTPA).^18^ The conjugation of DTPA to such polymers, followed by complexation with Gd^3+^ could result in an MR imaging contrast agent with high Gd^3+^ loading density and improved contrast enhancement.

**Fig. 1:**
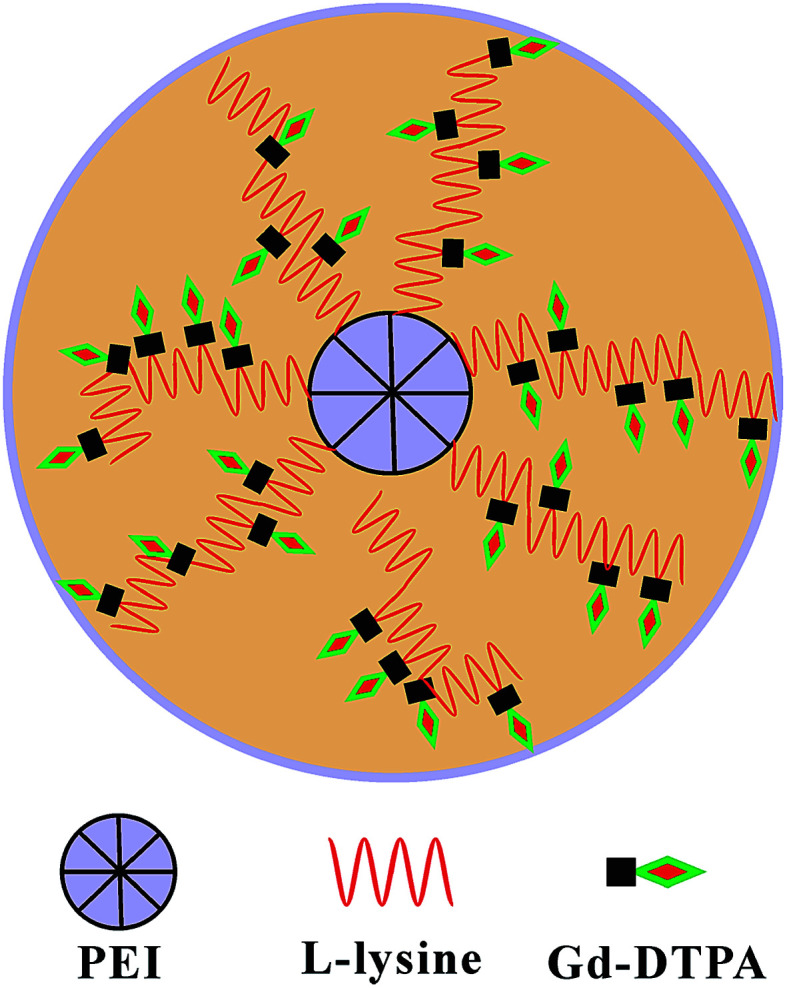
Schematic illustration of the design.

In this study, a star block copolymer PEI–PLL loaded with Gd^3+^ was designed and synthesized for MR imaging (Fig. 1). The star-block copolymer PEI–PLL–DTPA–Gd consists of a PEI core, PLL inner shell and large amount of Gd–DTPA. The synthesized PEI–PLL–DTPA–Gd was thoroughly characterized by different techniques and its capability for MR contrast imaging was evaluated both *in vitro* and *in vivo*. Results show that the synthesized PEI–PLL–DTPA–Gd displayed greater contrast and longer circulation time than conventional Gd–DTPA, and can be used as an enhanced *T*_1_ CA for blood pool imaging. Importantly, the facile synthesis and nontoxic properties of the polymers make it a highly potent CA for clinical applications.

## Materials and methods

### Reagents

Hyper-branched PEI (10 kDa), DTPA-dianhydride, Gd chloride hexahydrate (GdCl_3_·6H_2_O) and ε-benzoxycarbonyl-l-lysine (ZLL) were purchased from Sigma-Aldrich (St. Louis, MO, USA). The other synthetic reagents were purchased from Shanghai Jingchun Reagents Co., Ltd (Shanghai, China). All chemicals were analytical or higher grade. Dichloromethane (DCM), dimethyl sulfoxide (DMSO), dimethyl formamide (DMF) and ethyl acetate were dried over CaH_2_. Petroleum ether and tetrahydrofuran were dried by refluxing over sodium.

### Synthesis of materials

**Scheme 1:**
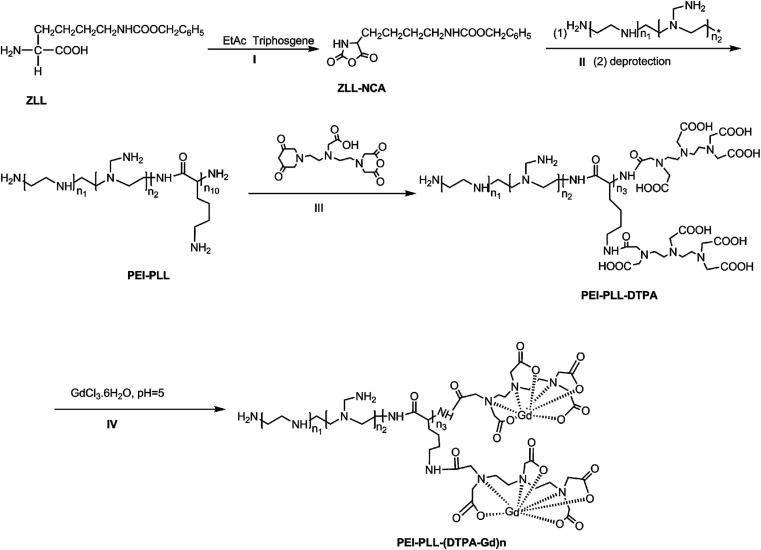
Synthesis route to PEI–PLL–DTPA–Gd.

PEI–PLL was synthesized (Scheme 1) according to the method published previously by our group.^14^ Briefly, ZLL–NCA was synthesized by phosphorylation of ZLL in anhydrous ethyl acetate (yield 70%), and then polymerized in anhydrous DCM with PEI as the macro initiator. An aliquot of a PEI stock solution in anhydrous DMSO (5 mL) was added to a solution of ZLL–NCA in DCM (50 mL). The reaction mixture was stirred at 25 °C for 12 hours. The resultant star polymer PEI–PZLL was isolated by precipitation in diethyl ether and dried under vacuum (yield: 90–93%). The ε-benzyloxycarbonyl group in PEI–PZLL was deprotected. Then, the PEI–PZLL was dissolved in trifluoroacetic acid and stirred for 30 min, followed by addition of anisole and methane sulfonic acid, and the resulting mixture was stirred at 25 °C for another 2 hours. The solution was diluted with fresh water and washed twice with diethyl ether to remove anisole. NaHCO_3_ solution (5.0 wt%) was added to the aqueous phase until a neutral pH was reached. The solution was then dialyzed by use of dialysis tubing [molecular weight cut-off (MWCO) 8–14 kDa, Union Carbide Corp. Chicago, IL, USA] and lyophilized with a lyophilizer (Labogene, Denmark) to obtain the final star-block copolymer PEI–PLL (yield: 80%).

PEI–PLL–DTPA was synthesized by an EDC (1-ethyl-3-(3-dimethylamino propyl) carbodiimide hydrochloride) coupling reaction.^19^ Initially, PEI–PLL was dissolved in 0.2 mol L^−1^ NaHCO_3_/Na_2_CO_3_ buffer solution (pH 9.6) in an ice bath, and then DTPA-dianhydride was added under stirring. After 16 hours, the product was dialyzed against water for 3 days, and then lyophilized to obtain the final products PEI–PLL–DTPA. Then PEI–PLL and PEI–PLL–DTPA were characterized by proton nuclear magnetic resonance (^1^H-NMR, DRX 400 MHz, Bruker, Germany). The molecular weight of PEI–PLL and PEI–PLL–DTPA was determined on a Waters 410 GPC system equipped with a 2414 RI detector (Waters, USA). An amount of 0.5 mol L^−1^ HAc–NaAc buffer (pH 4.5) was used as the eluent at a flow rate of 1 mL min^−1^ at 30 °C.

For chelation of Gd^3+^ ions on the block copolymer,^20^ the PEI–PLL–DTPA NPs formed were resuspended in water, and equimolar amounts of GdCl_3_·6H_2_O (1 mM) were added drop wise. The mixture was stirred for 1 hour for complete conjugation. The solution was dialyzed against water until no free Gd^3+^ was detected in the outside dialysis solution, and then the product was lyophilized. Then the Gd^3+^ content of PEI–PLL–DTPA–Gd was determined by the following method. PEI–PLL–DTPA–Gd was digested with aqua regia at room temperature for 24 hours and completely clear solution was obtained. The solution was then diluted to 10 mL with ultra-pure water. Then the gadolinium concentration of the PEI–PLL–DTPA–Gd was determined by inductively coupled plasma atomic emission spectroscopy (ICP-AES), carried out on a ICPE-9000 spectrometer (Shimadzu Corp., Japan).

### Characterization techniques

The morphologies of PEI–PLL–DTPA–Gd NPs were observed by transmission electron microscopy (TEM) (JEM-1200EX; Jeol, Japan). A droplet of sample was deposited on a carbon-coated 200-mesh copper grid to enhance the contrast. After 1 min, excess liquid was removed using filter paper. The grid was stained with 3% phosphotungstic acid solution and allowed to dry for TEM analysis. The size of the NPs, polydispersity index (PDI), and zeta potential were measured by dynamic light scattering (DLS) (Zetasizer Nano ZS90, Malvern, UK). All analyses were repeated in triplicate. The results are expressed as mean ± SD (*n* = 3).

### Chelate stability of PEI–PLL–DTPA–Gd NPs

In order to ensure good tolerability *in vivo*, the chelate stability of the proposed NPs was first evaluated *in vitro* by dialysis method and then ICP-AES measurements. Additional experimental details can be found in the (ESI†).

### Cytotoxicity of the PEI–PLL–DTPA–Gd NPs

The methyl thiazolyl tetrazolium (MTT) assay was performed to evaluate the cytotoxicity of the PEI–PLL–DTPA–Gd NPs. HepG2 cells, obtained from the American Type Culture Collection (ATCC, Rockville, MD, USA), were inoculated into 96-well plates at a density of 1 × 10^4^ cells per well. 24 hours later, the medium was replaced with PEI–PLL–DTPA–Gd NP suspensions at concentrations of 10, 20, 40, 80 μM. The commercially available contrast agent, Gd–DTPA, was used for the control treatment at a concentration of 40 and 80 μM. Cells with culture medium were chosen as the negative control. After incubation for 24 hours at 37 °C and 5% CO_2_, the two Gd formulations were removed and cytotoxicity was quantified by determining the viability of treated cells relative to negative control (negative control viability = 100%). Cytotoxicity results were therefore reported as relative viability, and that a higher relative viability was indicative of less toxicity.

### *In vitro* MR imaging

*In vitro T*_1_-weighted MR imaging and *T*_1_ relaxation rate were measured using a conventional fast spin-echo acquisition on a small-animal 7.0 T scanner (Agilent, US) with a 95/63 mm quad birdcage coil and gradient strength up to 400 mT m^−1^. *T*_1_-weighted MR images of the PEI–PLL–DTPA–Gd NPs and the Gd–DTPA injection were obtained at 23 °C in distilled water. MR images were taken at different concentrations of Gd^3+^ (0.016–2.0 mM). Samples were tested using *T*_1_-weighted pulse sequences. The sequence parameters were: TR, 800 ms; effective TE, 6.3 ms; echo train length, 8; matrix, 128 × 128; field of view, 50 × 40 mm; slice, 1; thickness, 2 mm; and average, 1. The inversion times were 10, 22, 51, 115, 260, 587, 1328, and 3000 ms. The *T*_1_ fitted value was obtained by using *T*_1_ mapping software of VnmrJ 4.0, a post-processing platform for the Agilent 7.0 T MR scanner. The *T*_1_ at different concentrations ([Gd^3+^] = 0.016–2.0 mM) was calculated. The *T*_1_ relaxation rate for PEI–PLL–DTPA–Gd and Gd–DTPA were calculated by the formula:1
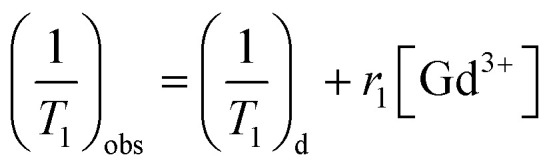
where (1/*T*_1_)_d_ is the relaxation time in the absence of the paramagnetic species, (1/*T*_1_)_obs_ is the relaxation time in the presence of the paramagnetic species, and *r*_1_ is the specific relaxivity.^15^ The correlation coefficient for the fitted line from three plots was 0.990, which was higher in all measurements.

### *In vivo* blood pool and major organ MR imaging

Kunming mice were provided by the Experimental Animal Center of Shantou University Medical College. All animal test procedures were performed in accordance with the National Institutes of Health guidelines (NIH Publication, revised 1996) on the use of animals in research, and were approved by the Institutional Animal Care and Use Committee of Shantou University Medical College. Mice were anesthetized with 2% isoflurane, in O_2_, delivered using a Summit Anesthesia Solutions vaporizer at a flow rate of 0.8 L min^−1^. Respiration rate was maintained at 25–30 respirations per min and monitored using a Biopac System MP 150. The *T*_1_-weighted images were performed using a small-animal 7.0 T scanner (Agilent, US). The parameters of *T*_1_-weighted pulse sequences were: TR, 800 ms; TE, 6.3 ms; matrix, 128 × 128; field of view, 60 × 60 mm; slice, 12; thickness, 2 mm. PEI–PLL–DTPA–Gd was injected (0.05 mM Gd per kg) through the tail vein. Two-dimensional spin echo MR images were obtained before and after administration of the materials, at 10, 30, 60, 180, 360 min and 24 h. The MR imaging signal intensity was measured using ImageJ (US National Institutes of Health, Bethesda, MD, USA). The contrast enhancement ratio (CER) for tissue or vessels was calculated according to the equation:2
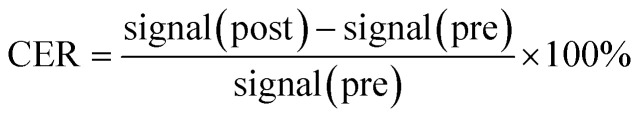


### Biodistribution study

Due to the low-sensitivity of the MRI to low concentration contrast agents, ICP-AES was performed on solution prepared by dissolving the major organs in aqua regia after 1 hour, 6 hour, 24 hour and 48 hour injection to evaluate the residue of the gadolinium uptake.^22^ And additional experimental details can be found in the ESI.†

## Results and discussion

### Synthesis of PEI–PLL–DTPA–Gd NPs

**Fig. 2:**
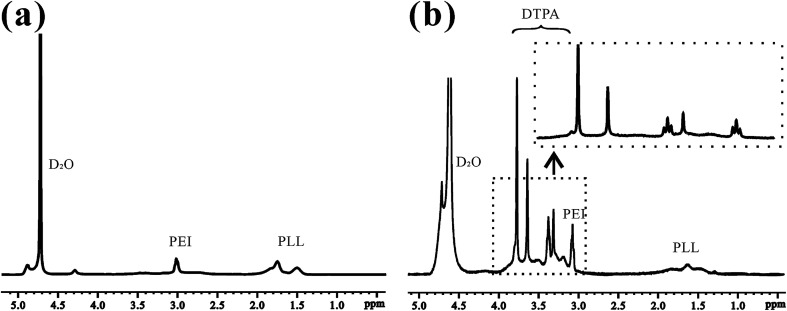
^1^H-NMR spectra of (a) PEI–PLL and (b) PEI–PLL–DTPA in D_2_O.

This is the first evaluation of the star-block copolymer PEI-PLL as an MR imaging CA carrier. The synthetic procedure for PEI–PLL–DTPA–Gd NPs is shown in Scheme 1. The primary amino groups of PEI were used as an initiator in the anionic ring-opening polymerization of Lys(Z)–NCA. The protected benzyloxycarbonyl groups in PEI–PLys(Z) were then removed using anisole and methane sulfonic acid to obtain PEI–PLL. The ^1^H-NMR spectrum of PEI–PLL is shown in Fig. 2a, and PEI–PLL–DTPA is shown in Fig. 2b. In the ^1^H-NMR spectrum of PEI–PLL, the peaks at 2.9 ppm and 1.2–1.9 ppm can be assigned to PEI and PLL, respectively (Fig. 2a).^23^ The chemical shift at 3.2, and 3.4 ppm can be assigned to the –N–C**H**_2_–C**H**_2_–N– of DTPA, and peaks at 3.6 and 3.8 ppm can be assigned to the –N–C**H**_2_–COO**H** of DTPA (Fig. 2b).^24^ Thus, ^1^H-NMR demonstrated successful synthesis of PEI–PLL and conjugation of DTPA to PEI–PLL.

By using the GPC data, the number-average molecular weight (*M*_n_) of PEI–PLL and PEI–PLL–DTPA were calculated. The *M*_n_ of PEI–PLL and PEI–PLL–DTPA was 18 071 and 50 818 Da, respectively; which means the number of DTPA moieties per polymer was approximately 83.25. The shift to a larger molecular weight to some extent indicates the successful conjugation of the DTPA group.

The introduction of Gd^3+^ ions to DTPA involved the addition of GdCl_3_·6H_2_O to the block copolymer in distilled water. The concentration of gadolinium content of the NPs was 6.30% (w/w). It is essential to reduce the possibility of toxic side effects and the onset of nephrogenic systemic fibrosis induced by free Gd^3+^.

### Characterization of PEI–PLL–DTPA–Gd NPs

**Fig. 3:**
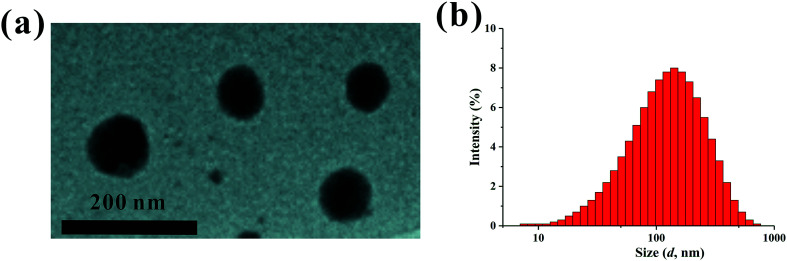
Transmission election microscopy (a) and dynamic light scattering (b) of the PEI–PLL–DTPA–Gd nanoparticles.

The PEI–PLL–DTPA–Gd NPs were spherical or ellipsoidal (Fig. 3a). No aggregation was observed, based on TEM. The PEI–PLL–DTPA–Gd NPs had a narrow size distribution and the mean size is 240.2 ± 15 nm measured by DSL (Fig. 3b) and 95 ± 17 nm measured by TEM by averaging the size of 100 NPs. The PDI of PEI–PLL–DTPA–Gd is 0.43 ± 0.0014 and zeta potential is 23.3 ± 0.8 mV.

### Chelate stability of PEI–PLL–DTPA–Gd NPs

Due to complicated *in vivo* environment of the existing of competing metal ions, the chelated Gd^3+^ may dissociate from the metal center by a transmetallation reaction. Since the free gadolinium is extremely toxic, the stability was assessed in the dialysis method. As shown in the Fig. S1 (ESI†), for using the mixture buffer as the outside solution, the gadolinium content within the dialysis bag was decreased during the first 2 hours and then the steady-state reached with about 83% gadolinium still in the dialysis bag. On the other hand, for using the pure PBS as the outside solution, the chelated gadolinium seems very stable without dissociation.

### Cell cytotoxicity

**Fig. 4:**
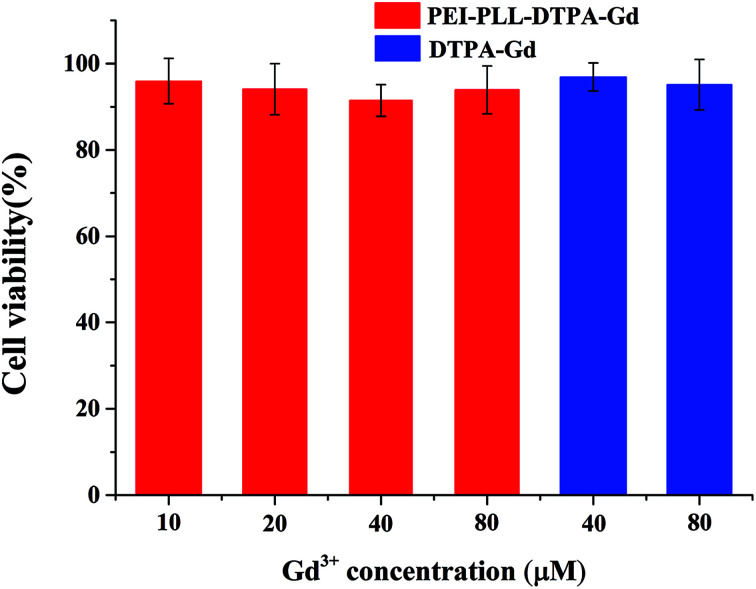
Cytotoxicity of PEI–PLL–DTPA–Gd nanoparticles on HepG2 cells.

*In vitro* toxicity of the PEI–PLL–DTPA–Gd NPs *vs.* Gd–DTPA at different Gd concentrations was evaluated by MTT assay using HepG2 cells (Fig. 4). Gd–DTPA at a high concentration of 40 μM and 80 μM were taken as a negative control. The cell survival following treatment with PEI–PLL–DTPA–Gd was slightly lower than Gd–DTPA. However, the cell survival of PEI–PLL–DTPA–Gd remained greater than 90% at all experimental doses examined, which suggests that PEI–PLL–DTPA–Gd NPs are safe to HepG2 cells at the tested concentrations. Compared with Gd–DTPA, toxicity experiment showed that the cell viability was not adversely affected over a wide range of PEI–PLL–DTPA–Gd.

### *In vitro* MR imaging

**Fig. 5:**
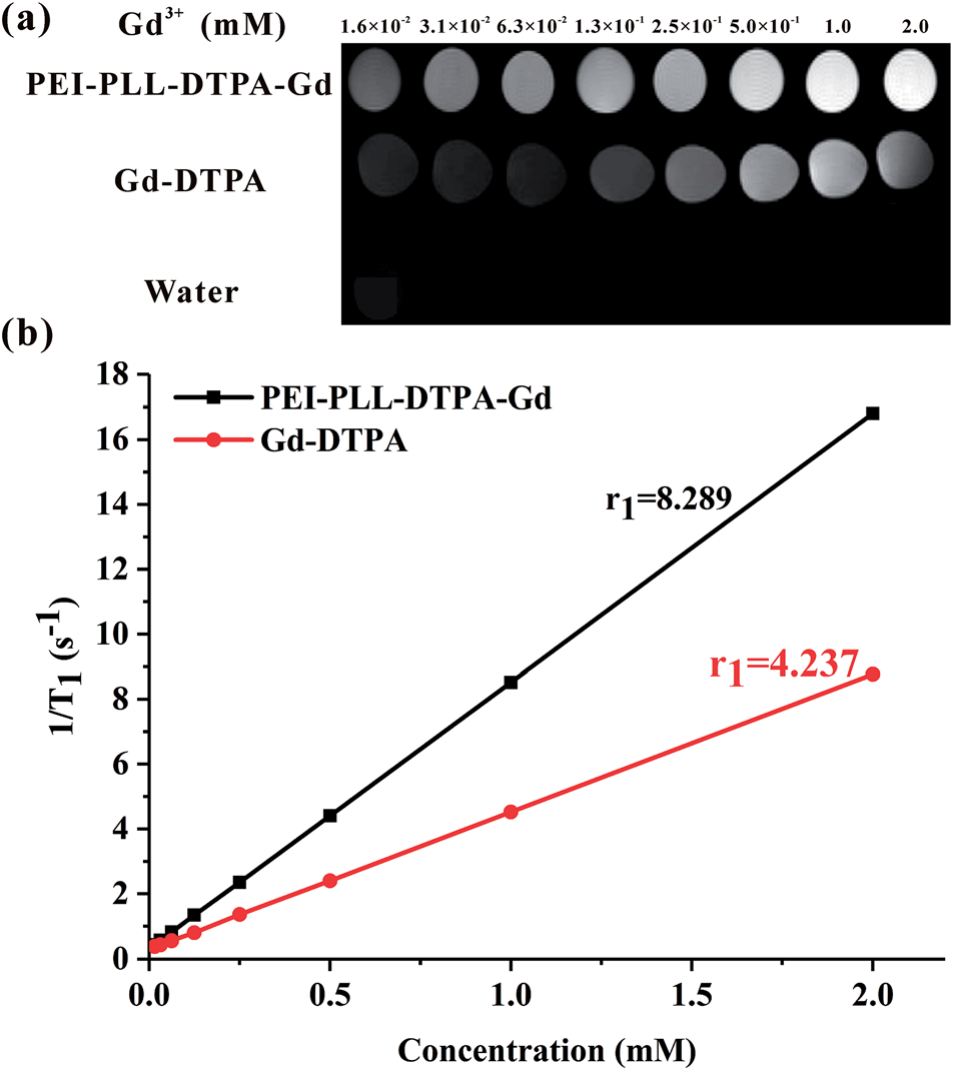
MR imaging results and relaxivity of PEI–PLL–DTPA–Gd NPs and Gd–DTPA.

To study the potential of PEI–PLL–DTPA–Gd NPs as an MR imaging contrast agent, the ability to shorten the *T*_1_ (longitudinal relaxation time) of water protons was measured. PEI–PLL–DTPA–Gd and Gd–DTPA were evaluated on a small-animal 7.0 T MR scanner. The concentrations ranged from 1.6 × 10^−2^ mM to 2.0 mM. For *T*_1_-weighted imaging, the higher Gd concentration of the NPs showed higher signal intensity. The PEI–PLL–DTPA–Gd signal (1.6 × 10^−2^ mM) was approximately equivalent to a Gd–DTPA injection of 2.5 × 10^−1^ mM (Fig. 5).

The relaxivity was calculated as the slope of the plot of 1/*T*_1_*vs.* concentration. The *T*_1_ relaxivity of PEI–PLL–DTPA–Gd was 8.289 mM^−1^ s^−1^, and the relaxivity of Gd–DTPA is 4.273 mM^−1^ s^−1^. The higher relaxivity of PEI–PLL–DTPA–Gd is a desirable property. The efficiency of enhanced imaging may be greater PEI–PLL–DTPA–Gd than Gd–DTPA at the same Gd^3+^ concentration, which could increase the sensitivity of MR imaging. Additionally, the required dose for the MR imaging may be reduced due to this high relaxivity. A high relaxivity translates to similar contrast with a smaller dose of contrast agent, which significantly reduces systemic exposure to toxic Gd^3+^.

Possible explanations for increased *T*_1_ relaxivity of PEI–PLL–DTPA–Gd compared to Gd–DTPA include: (i) an increase in rotational correlation time by virtue of the attachment of the metal chelate to macromolecules, and (ii) an increase in the number of outer sphere-coordinated water molecules entrapped by the PEI–PLL.^21^ The relaxivity of Gd-based macromolecular MR imaging CAs can be improved by reducing the rotational motion in solution.^25^ Since the Gd–DTPA molecules were linked to a large NP, the rotational correlation time was increased correspondingly. In addition, Gd–DTPA was modified on the surface of the NPs. Therefore, conjugation to the new NP could considerably enhance the relaxivity of the chelate units based on the increased size of the NP. The *in vitro* MR imaging results suggest that PEI–PLL–DTPA–Gd NPs could serve as an efficient MR imaging CA.

Without model calculation or nuclear magnetic relaxation dispersion (NMRD) experiments, we cannot claim the diameter of ∼200 nm is the most optimal size for changing the tumbling correlation time. According to previous studies,^26^ the size of the proposed nanoparticles was controlled in the range of ∼200 nm and the synthesis condonation for other size nanoparticles have not been explored in this piloted study. Generally, according to Stoke's law, molecular weight is positively correlated with its size. Interestingly, Lu *et al.* reported that two similar molecular weight PEGylated Gd–DTAP polymers had different *r*_1_.^27^ Recently, Decuzzi *et al.* found the tumbling correlation time seems critical for Magnevist to achieve high *r*_1_ but not for a gadolinium doped silicon particles. This may because beside the size of nanoparticles, the shape and surface properties will affect the *r*_1_ as well.^28^ With the help of NMRD to fitting proper parameters, it is meaningful to study mechanical behavior of size effect in the future and to obtain the most optimized size PEI–PLL.

The size of NPs plays a critical role, because it affects their physical and biological properties. In the previous studies, Li *et al.* synthesized a nanoscale micelle based on biodegradable poly(l-glutamic acid)-*b*-polylactide (PG-*b*-PLA) block copolymer with paramagnetic Gd^3+^ ions chelated to the shell. The average diameter of the copolymer is 230 nm and the *r*_1_ is 7.90 mM^−1^ s^−1^ on a 4.7 T scanner. The *r*_1_ is similar to that of PEI–PLL–DTPA–Gd, whereas the magnetic field is slight lower.^29^ Brougham *et al.* compared *r*_1_ of Gd-loaded-PAP (polychelating amphiphilic polymer)–liposomes and Gd–DTPA–BSA (bovine serum albumin)–liposome with almost equal hydrodynamic sizes (171 *vs.* 175 nm) and same fraction of Gd-bearing lipid. However, the NMRD results and *T*_1_ weighted images show a significant increase of *T*_1_ effect, and especially in the clinical B_0_ field range for the Gd-loaded-PAP but not for Gd–DTPA–BSA.^30^ Kotyk *et al.* proposed a lipophilic Gd–DTPA with size measured approximately 200 nm in diameter, which was linked the antibodies to the nanoparticles and the *r*_1_ is 12 mM^−1^ s^−1^ on a 4.7 T scanner. With different gadolinium concentration in the NPs, Gabor *et al.* found the *T*_1_ relaxivity of Gd–DTPA–PEI–NP 1 (160 nm) is 12.9 mM^−1^ s^−1^, which is smaller than that of same size Gd–DTPA–PEI–NP 2, but larger than that of proposed PEI–PLL–DTPA–Gd.^31,32^ To our knowledge, there was no systematic research on the relationship between macromolecular size and the longitudinal relaxation rate, which cannot be predicted by the Solomon–Bloembergen–Morgan (SBM) model. The large range of size controllable method for synthesizing polymers with different diameter may help to explore the mechanism in the future and to guide the research to optimize the size of nanoparticles for MR imaging enhancement.

### *In vivo* MR imaging

**Fig. 6:**
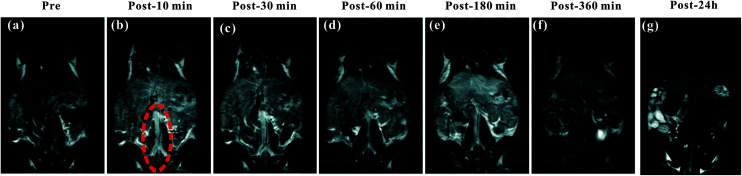
The *T*_1_-weighted MR images of a mouse injected with PEI–PLL–DTPA–Gd *in vivo*. (a–g) Pre-injection and 10, 30, 60, 180, 360 min and 24 h post-injection of PEI–PLL–DTPA–Gd, respectively. The abdominal aorta is indicated by red dashed circle in (b).

**Fig. 7:**
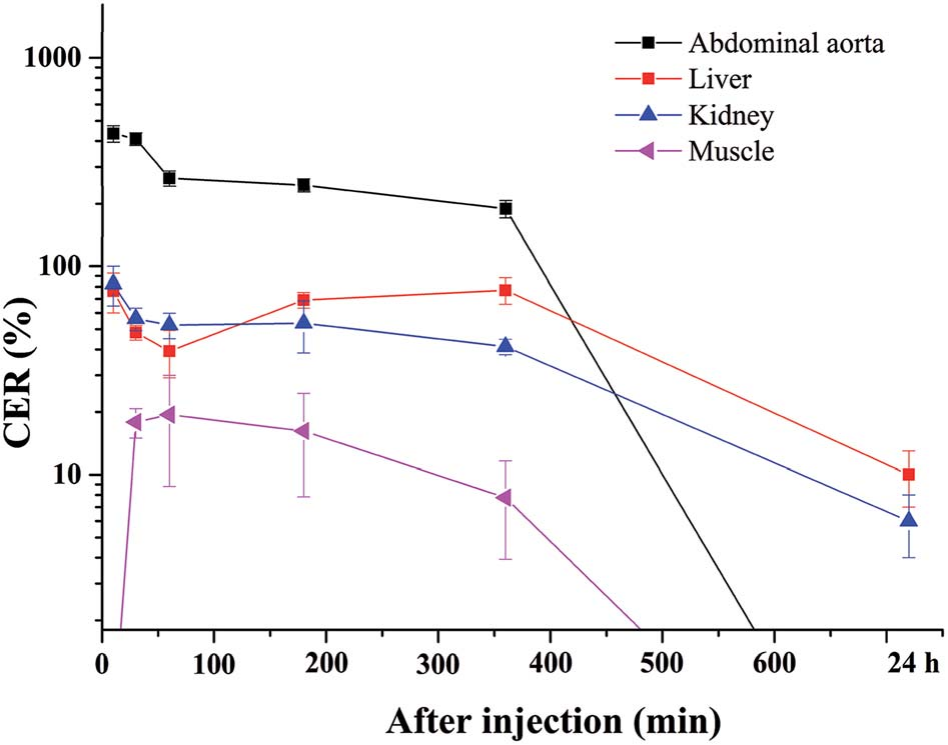
Quantitative signal intensity changes of regions of interest at 10, 30, 60, 180, 360 min and 24 h after injection of PEI–PLL–DTPA–Gd. The CER of the abdominal aorta, liver, kidney and muscle were shown.

The blood pool contrast enhancement (CE) capability of PEI–PLL–DTPA–Gd was examined by performing dynamic enhanced MR in Kunming mice (*n* = 3). Representative *T*_1_-weighted MR imaging scans (Fig. 6) show significant CE of a vascular system immediately after the injection of PEI–PLL–DTPA–Gd at a Gd dose of 0.05 mM kg^−1^ body weight. The CE of liver, vessels and heart were increased immediately after injection (Fig. 6), and significant CE of blood vessels persisted for up to 6 hours after post-injection. After 24 h, the enhancement disappeared for most tissues (Fig. 6g). For comparison, the *T*_1_ weighted Gd–DTPA enhanced *in vivo* MR images at different time points are shown in ESI (Fig. S3†). The time-enhancement changes of different tissues were show in Fig. 7.

Compared with Gd–DTPA,^33^ PEI–PLL–DTPA–Gd NPs had a significantly prolonged imaging time in the blood vessels, possibly because the large molecular weight of PEI–PLL–DTPA (>30 kDa) could make it harder to be excreted from the kidney.^15^ These results indicate that PEI–PLL–DTPA–Gd is suitable for serving as an MR imaging blood pool CA.

### Biodistribution study

Fig. S2† showed the biodistribution of gadolinium in the major tissues including heart, lung, liver, spleen, kidney and muscle. One hour after injection, gadolinium concentration was first measured in lung, liver, spleen and kidney and the spleen showed the highest concentration. The gadolinium concentration tended to decrease with increasing the time. On the other hand, no gadolinium was founded in heart and muscle.

## Conclusions

A new polymeric micellar MR imaging CA, synthesized by combing PEI–PLL star block copolymers and Gd, is proposed in this study. The reaction between PEI–PLL and a DTPA derivative results in quantitative DTPA conjugation regarding the lysine residues of the star block copolymer micelles. This micellar structure is maintained after partial chelation of the DTPA moiety with gadolinium ions. The viability of cells treated with Gd-conjugated PEI–PLL NPs is similar to cells treated with the clinically used Gd–DTPA contrast agent at the test concentrations. *In vivo* experiments confirm that the Gd-conjugated PEI–PLL NPs have a longer circulation time in blood vessels, compared with that of the conventional contrast agent Gd–DTPA. This polymeric micelle MR imaging contrast agent has the potential to be a useful diagnostic tool, particularly as a blood pool imaging CA.

## Conflicts of interest

There are no conflicts to declare.

## Supplementary Material

RA-008-C7RA08820E-s001
