# Supplementary material for: Gadolinium-conjugated star-block copolymer polylysine-modified polyethylenimine as high-performance T1 MR imaging blood pool contrast agents
Source: RSC Adv. 2018 Jan 30;8(9):5005–12. doi: 10.1039/c7ra08820e (PMC9078030; doi:10.1039/c7ra08820e)
Supplement: RA-008-C7RA08820E-s001 [file RA-008-C7RA08820E-s001.pdf]

Electronic Supplementary Material (ESI) for RSC Advances.
This journal is © The Royal Society of Chemistry 2017

Electronic Supplementary Information

Gadolinium-conjugated star-block copolymer polylysine-modified polyethylenimine as high-performance T_1 MR imaging blood pool contrast agents

Zhongjie Huang[1], Yicun Chen[2], Daojun Liu[3*], Chao Lu[3], Zhiwei Shen[4], Shuping Zhong[5] and Ganggang Shi [2*]

[1] Department of Radiology, the First Affiliated Hospital, Shantou University Medical College, Shantou 515041, China; E-mail: zjhuang@stu.edu.cn (for Zhongjie Huang)

[2] Department of Pharmacology, Shantou University Medical College, Shantou 515041, China; E-mail: chenyicun@yeah.net (for Author Yicun Chen), ggshi@stu.edu.cn (for Ganggang Shi)

[3] Department of Chemistry, Shantou University Medical College, Shantou 515041, China; E-mail: Liudj@stu.edu.cn (for Daojun Liu), sampson020@163.com (for Chao Lu)

[4] Department of Radiology, the Second Affiliated Hospital, Shantou University Medical College, Shantou 515041, China; E-mail: zwshen@stu.edu.cn (for Zhiwei Shen)

[5] Department of Biochemistry and Molecular Biology, Keck School of Medicine, University of Southern California, Los Angeles, California 90033, USA; E-mail: szhong@usc.edu (for Shuping Zhong)

Contents

1. Figure S1. Complex stability of PEI-PLL-DTPA-Gd in a dialysis bag with the external solution of pure PBS and of a mixture buffer containing equal (7.77 mM) $MgCl_2$, $CuCl_2$ and $CaCl_2$ in PBS at pH 7.4 and 37 °C.
2. Figure S2. The biodistribution of Gd^{3+} concentration of PEI-PLL-DTPA-Gd in mouse in different tissues.
3. Figure S3. The T_1 weighted Gd-DTPA enhanced *in vivo* MR images at different time points.

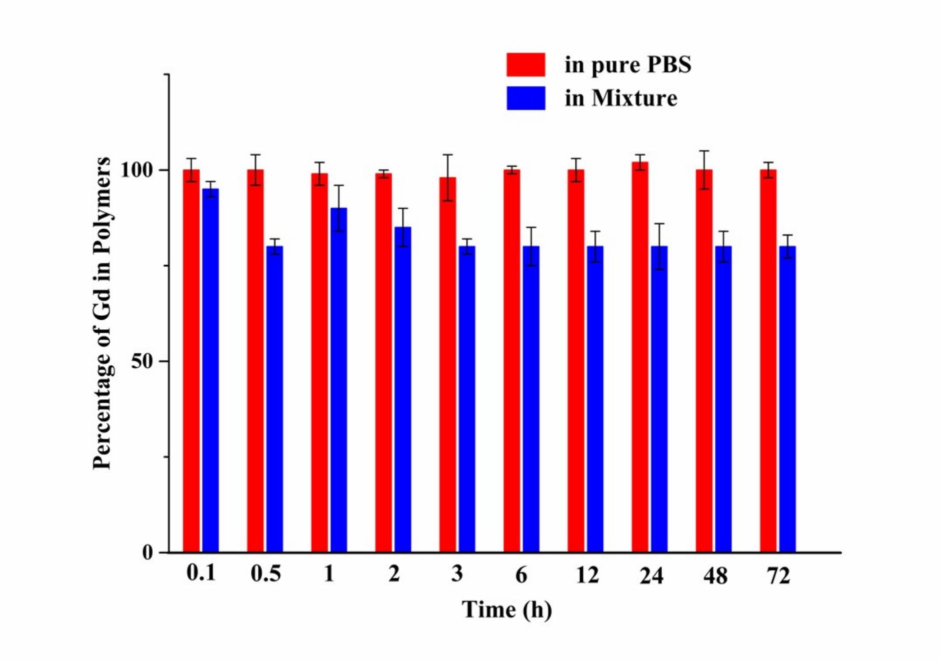

Figure S1. Complex stability of PEI-PLL-DTPA-Gd in pure PBS buffer in a dialysis bag with the external solution of pure PBS and of a mixture buffer containing equal (7.77 mM) $MgCl_2$, $CuCl_2$ and $CaCl_2$ in PBS at pH 7.4 and 37 °C. Three equal weight PEI-PLL-DTPA-Gd (0.1 g) was dissolved in 5 ml phosphate buffered saline (PBS) buffer, and then the solution was transferred into a dialysis bag with a molecular weight cut-off of 14 KD. The dialysis bag was putted in to a beaker with mixture of $MgCl_2$, $CuCl_2$ and $CaCl_2$ in PBS and only pure PBS as well. After 0.1, 0.5, 1, 2, 3, 6, 12, 24, 48 and 72 h, 1 ml solution in the dialysis bag and beaker was pipetted out for ICP-AES measurements and equal volume pure PBS was replenished.

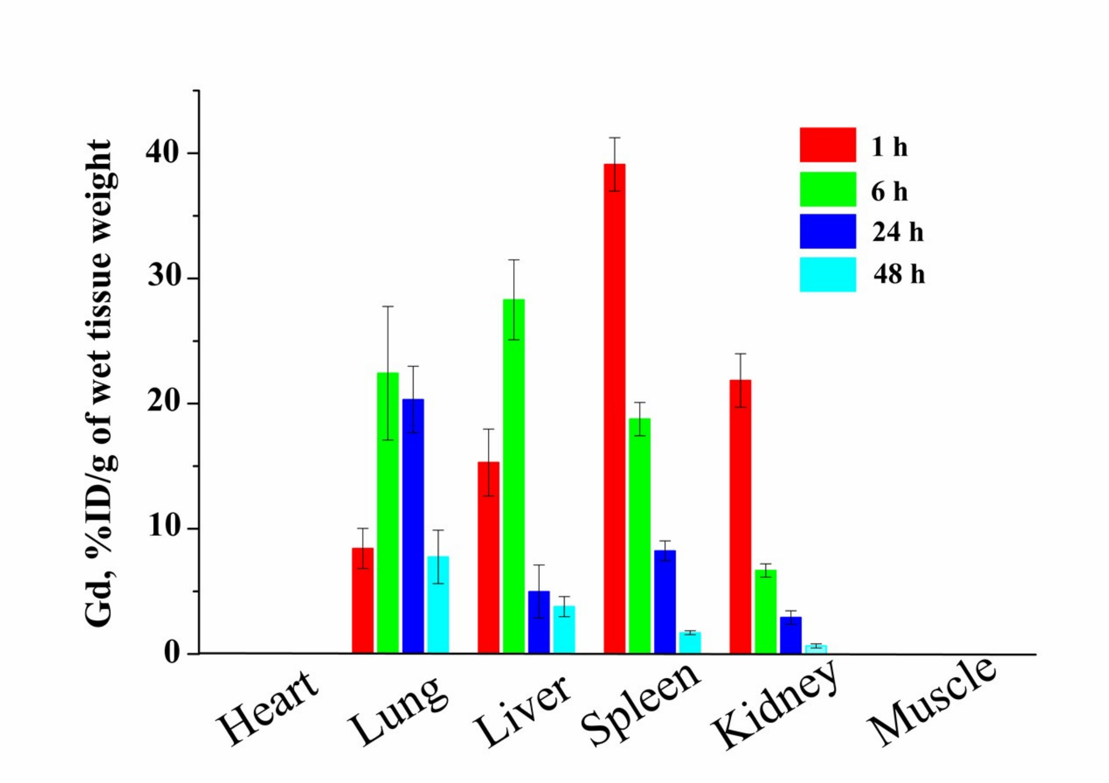

Figure S2. The biodistribution of Gd^{3+} concentration of PEI-PLL-DTPA-Gd in mouse in different organs (n = 3). Mice were injected with PEI-PLL-DTPA-Gd through the tail vein (0.05 mM/Kg), and then three mice were sacrificed with an overdose of isoflurane at each time point. After that, sever tissues including heart, lung, liver, spleen, kidney and muscle were extracted and weighted. Then the tissues were digested with aqua regia to obtain clear solution before ICP-AES measurements. The Gd^{3+} concentration was expressed as the percentage of the injected dose per gram of wet tissues weight (%ID/g).

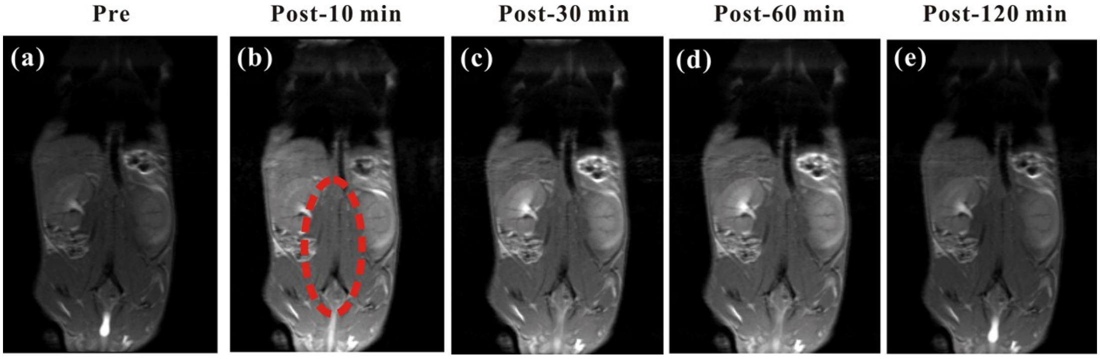

Figure S3. The T_1 weighted Gd-DTPA enhanced *in vivo* MR images at different time points.
